# Assessing the cost barrier for small and medium food processing businesses to meet Preventive Controls for Human Foods standards

**DOI:** 10.1371/journal.pone.0306618

**Published:** 2024-09-13

**Authors:** Jill Ann Fitzsimmons, Amanda J. Kinchla, Christina Allingham

**Affiliations:** 1 Department of Resource Economics, University of Massachusetts Amherst, Amherst, Massachusetts, United States of America; 2 Department of Food Science, University of Massachusetts Amherst, Amherst, Massachusetts, United States of America; University of Agriculture Faisalabad, PAKISTAN

## Abstract

The Preventive Controls for Human Food Rule regulation under the Food Safety Modernization Act sets new food safety standards. Both food safety educators and small and medium sized food processing businesses that manufacture certain value-added or processed foods lack knowledge regarding costs to prepare, implement, and manage ongoing food safety practices under the new standards. Current food safety training materials do not acknowledge costs or provide content addressing potential costs, and food safety educators do not have information needed to guide development of relevant materials that address costs. We combine economics and food science principles and use mixed methods to identify and estimate cost barriers for food processing businesses through an interdisciplinary research and extension project in the Northeast U.S. We first modify Preventive Controls extension programming to acknowledge costs and test how modified programming improves self-reported knowledge about costs. Materials that acknowledge that costs are associated with meeting standards significantly increases participants’ self-reported perceived knowledge of costs by 1.3 points on a 1-to-5-point Likert scale. Compared with programming areas in which detailed content is provided, however, improvements in knowledge of costs lags behind overall knowledge gains (3.2 for costs *versus* 4.1 for food safety content). To fill this content gap, we next conduct semi-structured group interviews with a subset of participants (N = 10), develop a costs analysis framework, and measure actual costs associated with Preventive Controls. We find that initial costs average $20,000 per business to plan, implement, and manage standards, and almost $8,000 in every subsequent year to manage. We demonstrate that even modest interventions can reduce cost barriers for businesses seeking to meet compliance standards. We provide food safety educators with concrete cost information to support businesses to pursue Preventive Controls standards. Study results imply that low-cost methods could improve food safety in mid-scale supply chains.

## Introduction

The Food Safety Modernization Act (FSMA) transitions food safety requirements from reactive actions to proactive practices [1]. FSMA, signed into law in 2011, implements a new foundational rule called “Current Good Manufacturing Practices Hazard Analysis and Risk-Based Preventive Controls for Human Food”, commonly known as the Preventive Controls for Human Food Rule (PCHF). This rule includes new standards and requirements for food businesses to identify and manage food safety preventive controls for certain processed foods not already covered by other U.S. federal food safety regulations (S1 File). PCHF creates a fundamental change in the U.S. regulatory landscape in which processors must pro-actively demonstrate compliance, either through filing a formal attestation with the FDA that they are exempt from meeting certain standards or by meeting standards and registering with the FDA [2]. Full regulatory compliance requirements for all processors went into effect in January 2020 [3].

PCHF increases food safety regulatory standards and requirements, but there is lack of clarity about which standards are required for which food businesses, and the process businesses must go through to meet requirements and demonstrate compliance. The standards that FSMA requires large food processors to meet are clear: Processors must develop and implement a complete, written, food safety plan (FSP) [3]. Smaller processors, however, face a confusing regulatory landscape. They may be exempt from completing a full written FSP, but are still required to meet some of the standards and are encouraged to meet full standards. FSMA determines which food processors are required to meet which standards based on size and marketing scope, where “Qualified-Exempt Facilities” are not required to comply with all standards of the PCHF Rule [4]. Qualified Exempt Facilities include very small food businesses “averaging less than $1 million per year (adjusted for inflation) in annual sales of human food plus the market value of human food manufactured, processed, packed, or held without sale” and small food businesses “with fewer than 500 full-time equivalent employees” [5]. Businesses that do not meet the definition of “very small” or “small” businesses can still be a qualified facility if the annual average of all food sold is less than $500,000 and at least half of that food is sold to “qualified end users” who live within 275 miles of the facility [2, 4]. The FSMA regulatory guidelines do not provide a formal definition of this group; We refer to these businesses as “medium-sized”.

While very small, small, and many medium-sized processors (SMPs) are Qualified Exempt Facilities, they must still comply with general food safety and sanitation standards within Current Good Manufacturing Practices, identify and control the food safety hazards associated with their products, and comply with local and state food safety regulatory requirements [6]. SMPs that choose to comply with PCHF under the Qualified Exempt status must file an attestation with the FDA that documents and acknowledges the grounds on which they are exempt [4]. Regardless of whether businesses are responsible for meeting all new standards under federal regulations, food safety educators encourage SMPs to proactively implement compliance tasks to meet the best practices in food safety that are outlined in the new standards. In addition, food businesses face increasing demands from downstream markets to undertake compliance tasks to fully meet the standards set by PCHF for the purposes of obtaining a third-party audit, even if they are not required to. Because of this, some SMPs that are eligible to comply with PCHF as Qualified Exempt Facilities may instead chose to fully meet the new standards and file with the FDA as what we refer to as “fully compliant”, *i.e.* under the same standards required for large processors. Other SMPs may choose to partially meet the full requirements by undertaking some compliance tasks and achieving some standards.

Despite the many reasons to implement compliance tasks and meet the new standards identified in PCHF, the perceived costs of compliance tasks prevent SMPs from attempting to meet new standards. Like businesses that are required to comply with environmental, labor, or building standards, SMPs are heterogeneous in both their attitude toward the costs/benefits, and their awareness of actual costs/benefits. Baron and Baron [7] identified that businesses comply with regulations when either the *perceived* marginal benefit of compliance or the *perceived* marginal cost of non-compliance are equal to or greater than the *perceived* marginal cost of compliance. Henson and Heasman [8] expanded on the concept of “perceived” marginal costs and benefits to model the stages of food safety compliance, beginning with the firms’ awareness of the regulations through eventual implementation and monitoring/evaluation (S1 Fig). Henson and Heasman [8] highlight that, when firms reach the “Compliance Decision” stage, “firms’ perceptions of the cost-benefit relationship associated with regulations is as important as a recognition of the actual costs and benefits involved, since it is the former that will actually drive business decisions”. To address this consideration in the case of food safety regulatory compliance, Henson and Heasman surveyed food business firms in the U.K. (N = 67) regarding both how easy it is to identify costs of food regulation compliance, and how easy it is to quantify the costs of food regulation compliance [8]. They find that over 65% of respondents found it either “Difficult” or “Very Difficult” to identify costs, and over 77% found it either “Difficult” or “Very Difficult” to quantify costs.

Henson and Heasman point out, that “since benefits are generally more difficult to quantify than costs, there will tend to be an in-built bias towards relatively higher perceived costs” [8]. Businesses are therefore more likely to expect that the costs of compliance are higher than the benefits, thus making decisions on the margin not to implement food safety measures. Subsequent studies underscore their findings that costs are critical barriers to food safety compliance. In the U.K., Mensah and Julien [9] found that costs are a top-five challenge and Yapp and Fairman [10] found that small and medium food enterprises perceived money to be critical barriers. In the US, Grover, Chopra and Mosher [11] found that cost of implementation was critical, due to cost of infrastructure investments, third party consultants, and employee training. Barone, DiCaprio and Snyder [12] similarly found that “cost” and “time” were the most frequently identified barriers for processors in Ohio. A survey of Food Safety Educators in the Northeast found that barriers related to learning Food Safety Content consistently ranked lower than costs-related barriers [13]. This suggests that there is a gap between what SMPs’ “perceive”, or fear, the costs of compliance may be, and evidence-based knowledge of what the costs actually are, indicating that the cost barrier may be about more than the actual financial barrier of a compliant FSP. S2 Appendix contains detailed discussion of these studies. The FDA itself acknowledges that small food processors face cost barriers to implementing PCHF [14]. Negative aggregate impacts of cost barriers to food safety compliance can reduce competitiveness of the sector, and have been considered serious enough to constitute a non-tariff barrier to trade [15].

The FDA-approved PCHF trainings (created by the Food Safety Preventive Controls Alliance) [16], provide detailed instruction on food safety content, in particular the process to develop a PCHF food safety plan (FSP). The course does not, however, provide SMPs with information regarding costs associated with implementing the Rule, or even mention that costs are associated with implementation [12]. While perceived costs have been identified as a potential barrier to PCHF implementation, no research to date evaluates to what extent current Preventive Controls Qualified Individual (PCQI) training content effectively reduces the cost barrier. As such, the industry-standard training available to SMPs to be recognized as a PCQI, the PCHF standardized curricula, does not address one of the most important barriers to implementation.

The cost barrier to meeting PCHF standards is closely related to cost barriers that farmers face to implement FSMA’s Produce Safety Rule. Adalja and Lichtenberg [17] found that while agricultural producers feared Produce Safety Rule compliance would be prohibitively costly, the cost barrier decreased with farm size. Schmit et al. [18] found that despite concerns that Produce Safety Rule-related Good Agricultural Practices would create relatively larger barriers for smaller producers, gains from implementation offset the costs. Baron and Frattaroli [19] similarly found that small direct-market poultry producers identify time and energy as important barriers to food safety practices, in addition to scale-appropriate up-front capital costs.

To improve SMPs’ adherence to best practices in food safety as identified in PCHF, it is important to address costs associated with planning, implementing, and managing a PCHF FSP. Accordingly, this paper addresses two critical issues. First, we assess to what extent current knowledge and modified PCHF training content can increase SMPs’ knowledge regarding costs of PCHF compliance. Second, we establish a framework to identify and measure compliance costs and use this framework to provide baseline estimates for PCHF compliance costs for SMPs.

## Materials and methods

We combine economics and food science principles and use mixed methods to identify and estimate the barriers presented by PCHF compliance task costs in the context of an interdisciplinary research and extension project. In the project, we provided extension education and technical assistance programming. To evaluate whether modified PCQI content improved SMPs’ self-reported knowledge about costs, we measured pre- and post- self-reported knowledge of all participants at several stages of the extension programming. To develop a framework and measure actual costs associated with compliance tasks we conducted semi-structured group interviews with a subset of the participants at the end of the programming. Our population of interest includes food processing businesses in the Northeast US that are considered “Qualified Exempt Facilities” under FSMA’s PCHF rule, identified above as very small, small, and medium sized businesses.

### Extension and technical assistance programming

The extension education and technical assistance programming provided intensive, tiered training and technical assistance to SMPs in the Northeast. For many SMPs, the only available PCHF training is the PCQI training. The standard PCQI training is 2.5-day (20 hours) and is designed for food businesses that already possess baseline competencies that most SMPs do not have. The standard PCQI training does not provide non-Food Safety related content, such as content that addresses the costs of compliance tasks, including financial risks associated with non-compliance. SMPs that complete the PCQI training often struggle to implement the content that they have learned during the PCQI course [12].

Instead, our programming guided SMPs through the PCHF-compliant FSP development process in smaller steps. Altogether, we offered four educational program units that incrementally increased in detail and SMP commitment (Table 1). The first unit consisted of a low-stakes environment for SMPs—a short 45-minute informational webinar to provide SMPs with information about how to comply with PCHF. The next unit was a 3-hour virtual workshop that introduced key concepts within PCHF. Participants that attended the 1-hour webinar and the 3-hour virtual workshop were then invited to participate in the third unit, the modified full PCQI training. We extended the traditional 20-hour program content to 24 hours (3 full days) of contact hours to allow space for discussion and questions, and to deliver supplemental materials to provide additional support to the SMP audience. To enhance recruitment and alleviate the economic barrier of participation, we offered scholarships to attend the 3-day training for free.

**Table 1:** Description of extension and technical assistance topics by program unit.

**Course 1: One-hour Webinar**	**Course 2: Three-hour Workshop**
Overview of Preventive Controls	What is HACCP?
FSMA	GMPs
Preventive Controls Rule	Contents of a FSPs
FSP	Hazard Analysis
Hazard Analysis	Preventive Controls
Legal Implications	Modified Requirements
Costs	Attestations for Qualified Processors
Recommendations for future planning	
Resources for planning	
**Course 3: Supplemented Three-day Preventive Controls Qualified Individual (PCQI) Training Course**	**Course 4: Working Group Sessions**
Introduction to the FSPCA Human Food Course	Preliminary Steps (process & product desc., process flow diagram, costs)
FSP Overview	Hazard Analysis Development
Costs and Risks	Identification of Preventive Controls, Validation and Verification
Good Manufacturing Practices & Prereqs	Records, Implementation and Monitoring
Biological FS Hazards	Auditing
Chemical, Physical, Economic FS Hazards	Environmental Monitoring Programs & Costs
Preliminary Steps in Developing a FSP	
Resources for Preparing FSPs	
Hazard Analysis & Preventive Controls Determination	
Process Preventive Controls	
Food Allergen Preventive Controls	
Sanitation Preventive Controls	
Supply-Chain Preventive Controls	
Verification & Validation Procedures	
Record-Keeping Procedures	
Recall Plans	
Regulation Overview	
Environmental Monitoring Programs	

Table notes Food Safety Plan (FSP)

The final unit directly engaged SMPs in online Group Sessions over the course of three months to develop and implement their own respective FSPs. The SMPs divided into two groups; one group (Group 1) received additional consultation and assistance. Group 1 worked directly with food safety experts in an online format. These online sessions broke FSP development into six sections over three months (preliminary steps, hazard analysis, PC identification, *etc.*). Each processor in Group 1 worked to implement compliance tasks to and complete their own FSP that reflected the standards they met for their specific processing environment, and the food safety experts provided one-on-one feedback on their “homework” assignments. The food safety experts also conducted site visits to all Group 1 facilities. Most of the SMPs in Group 1 had an end goal to pass a third-party audit of their facility, which required meeting all or almost all PCHF standards. Group 2 was hosted in a similar online format but did not include one-on-one FSP feedback from food safety experts or site visits to their facilities. Group 2’s sessions covered the same content as Group 1’s. Group 2 received feedback in the context of online group discussions.

The end goal of the programming differed depending on the needs of each individual SMP. For some SMPs, the end goal was to fulfill the minimum compliance tasks required to meet the standards as a Qualified Exempt Facility. Other SMPs aimed for “full compliance” with the PCHF to pass a third-party audit to satisfy demands of downstream buyers, despite being eligible to be a Qualified Exempt Facility. Many SMPs aimed for the middle ground—satisfying the regulatory requirements and achieving as many compliance tasks as they were able to.

### Recruitment methodology and goals

While SMPs are not technically considered to be “Human Subjects” for the purposes of Institutional Review Board (IRB) standards, we opted to design and submit for approval an IRB research protocol for our project (Protocol #2205 & #2036). We made this decision because we intended to ask participants for potentially sensitive financial information about their businesses, and we wanted to ensure that they felt safe and confident sharing accurate information [20]. Authors had access to information that could identify individual participants during and after data collection.

Our recruitment efforts deliberately targeted small and medium processors as a unified participant pool, forming a cohesive cohort. The FDA does not formally define “medium” processors as a regulatory sub-group, and the “Qualified Facility” status applies equally to both sub-groups. Given the intricacies of engaging with processors under a relatively new regulation, this approach stemmed from our concern about the challenges associated with reaching this specific audience. There is uncertainty regarding processors accurately determining their business size for compliance purposes. Additionally, we were interested in processors generally classified as qualified exempt from the full Preventive Controls for Human Food (PCHF) rule.

We first recruited participants for the webinar from a broad pool of SMPs in the Northeast US, with a target of enrolling 111 SMPs. From the SMPs that attended the webinar, our goal was to recruit 53 SMPs to attend a workshop. From those that attended a workshop, our goal was to recruit 20 SMPs to attend the three-day PCQI course. Finally, we targeted a goal of recruiting four participants to participate in Group 1 and four participants to participate in Group 2. The tiered design reflects both the increasing intensity of time and commitment on the part of participants, and the increasing time and effort required for our food safety team to provide high-quality training and technical assistance.

**Fig 1:**
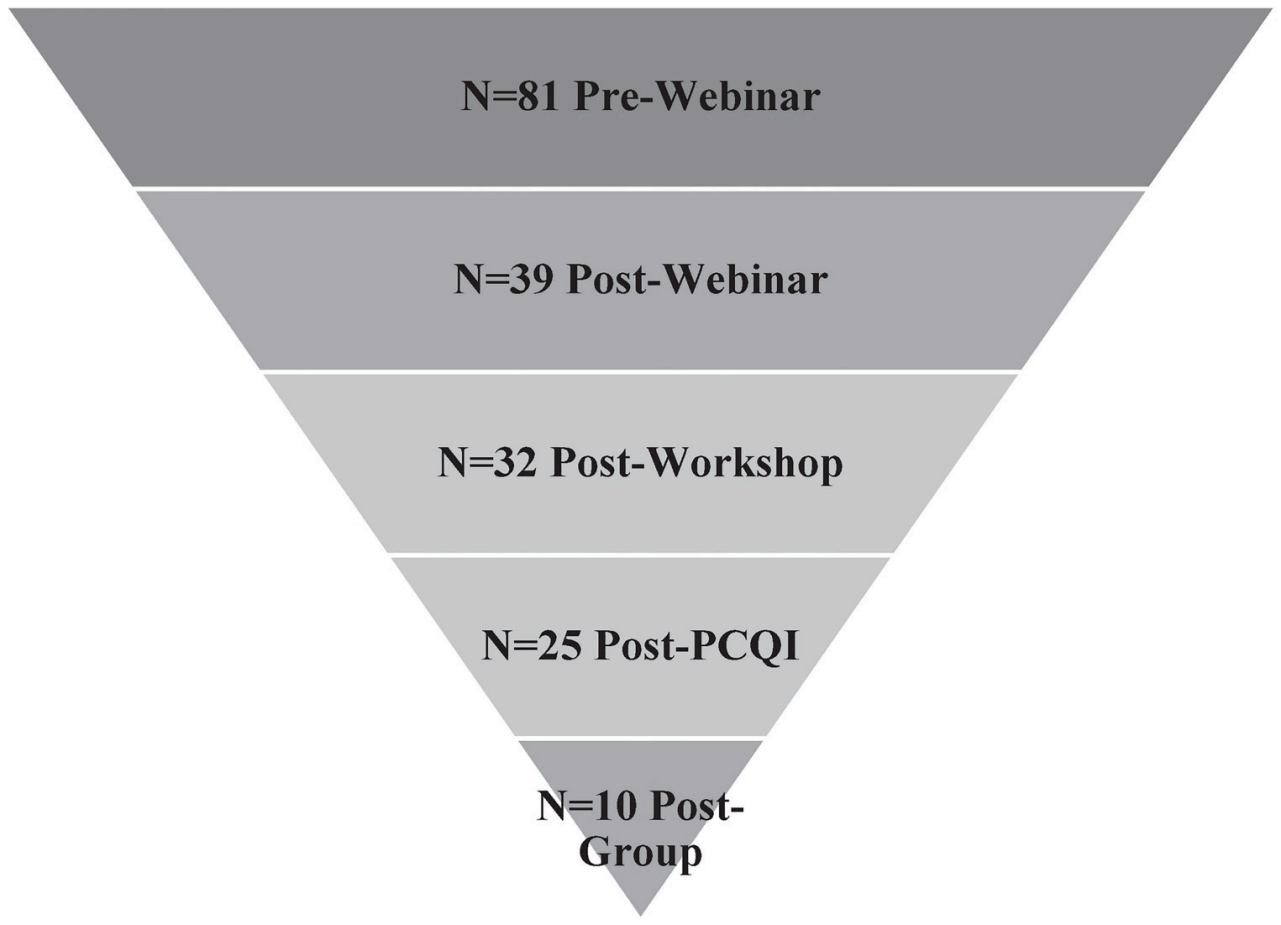
Participants per program unit.

In the summer of 2020, we compiled a list of 1,517 email addresses from SMPs in the Northeast U.S., primarily through internet searches. We designed a short webinar registration and pre-evaluation form using Qualtrics LLC survey software and distributed webinar invitations to our list. We offered four webinar sessions in the late summer and fall of 2020. Of the email invitations sent, 319 emails were bounces, duplicates, or opt-outs. After several reminders, 81 SMPs registered for one of the four webinars with a response rate of about 6.7%. While this initial recruitment was lower than our target goal, our subsequent retention met or exceeded our recruitment goals. About 48% of Webinar attendees took the next step and attended a 3-hour online PC Workshop, and about 40% of the Webinar attendees (and 82% of the Workshop attendees) attended a three-day PCQI course. Of these, the interest in participating in the Group sessions was very high, and we enrolled our target number of four SMPs in Group 1 and increased our Group 2 enrollment to accommodate six SMPs. The retention rates between the tiers of technical assistance programming indicate that SMPs that engage with PC Resources are highly likely to recognize the importance of pursuing PCHF compliance and take steps to achieve compliance. The very low initial response rates, however, indicate the steep challenges to engaging SMPs in the first place. There were 39 participants in the Workshop, 25 participants in the PCQI course, and 10 participants in the Group Sessions, shown in Fig 1. We obtained written informed consent from each participant. Additional details regarding study design are provided in S1 Appendix.

### Knowledge framework

**Fig 2:**
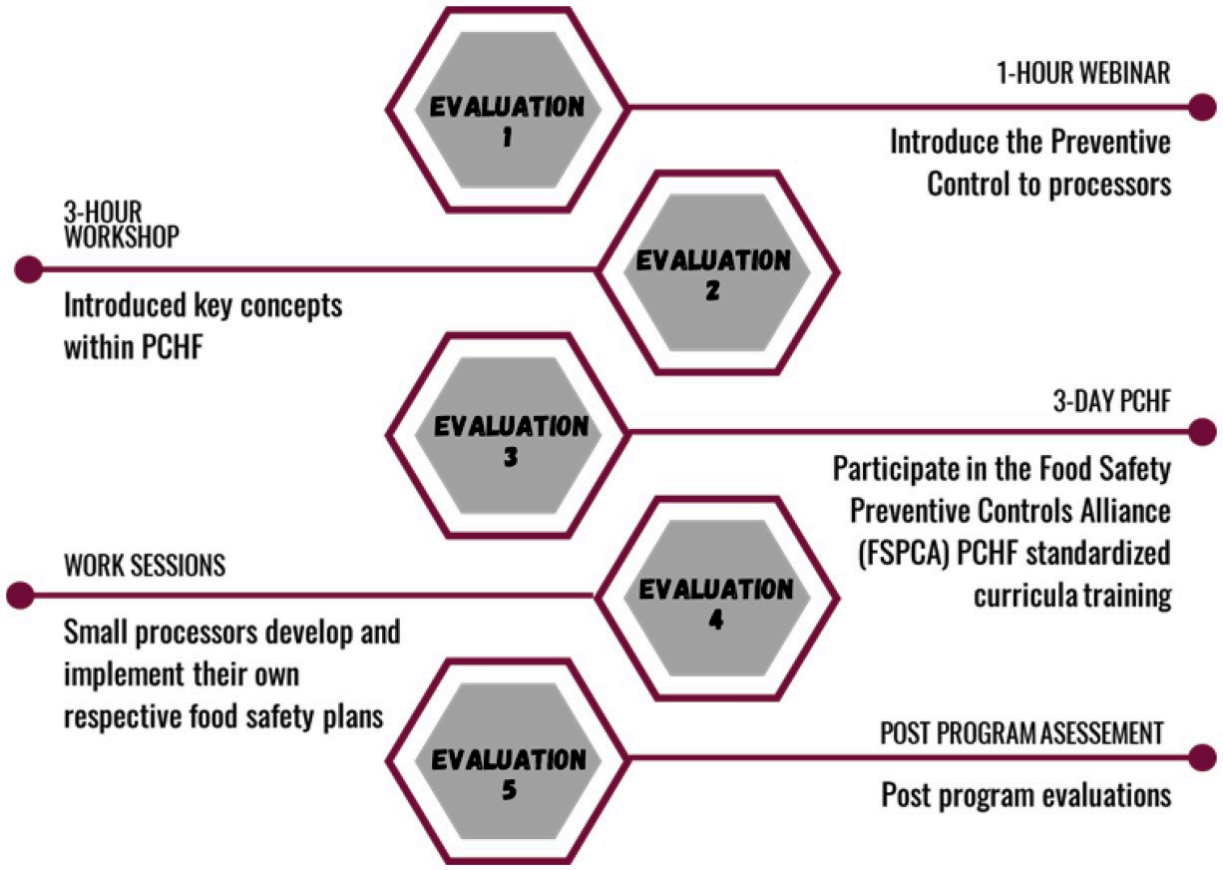
Programming and evaluation overview.

To test changes in perceived knowledge, we evaluated participants five times throughout the programming: once prior to the educational programming, and again after each educational unit Fig 2. We asked participants to rate their knowledge in fifteen areas of FSP planning and implementation on a 5-point Likert scale from “Not knowledgeable at all” to “Extremely knowledgeable” S1 Table. Most learning areas that we asked participants to rate correspond to learning goals in the PCHF standardized curricula. We added, however, three non-required areas that covered non-Food Safety content, including “Estimating costs of compliance and financial risk of non-compliance” and “Filing an Attestation” and “Audit Preparedness”. Completing the evaluations was required for each SMP to advance to the next unit. Data were downloaded from the online survey and cleaned. Descriptive statistics were prepared in Microsoft Excel, while analysis for statistical difference between programming units was performed using two-tailed unpaired unequal T-tests for each knowledge area in Stata 16.0. Our null hypothesis is that for each knowledge area there are no statistically significant differences in mean reported knowledge in between the first programming unit Pre-Webinar, *μ*_*pw*_, and the fourth programming unit Post-PCQI, *μ*_*pp*_.

$$\begin{array}{c} H_{0}:\mu_{pw}=\mu_{pp} \end{array}$$ (1)

$$\begin{array}{c} H_{A}:\mu_{pw}\neq \mu_{pp} \end{array}$$ (2)

### Costs framework

To develop a framework to identify and measure costs and obtain baseline costs estimates, we conducted semi-structured focus group interviews [20]. Group interviews were conducted virtually via Zoom due to the COVID-19 Pandemic. Participating SMPs included those that attended the entire programming sequence, culminating in the Group Sessions, over the course of about eight months. We held two interview sessions in which participants were assigned to a group based on whether they had been in Group 1 or Group 2. The same questions and visual prompts were used in both interviews. In the interviews, we asked the SMPs to consider only one of the products manufactured. Details regarding qualitative methods used are provided in S1 Appendix.

To understand costs that SMPs face, we consider the different stages of PCHF-compliant FSP preparation. We first consider the Planning Stage of FSP preparation. This stage involves the learning and planning that SMP owners and managers must undertake to prepare the written FSP. The Planning Stage, of course, will look different for new businesses, or businesses that are in the process of a major shift in production due to scaling up or facility changes, compared to established businesses that need to modify existing processes. In this project, all of our participants were existing SMPs with baseline food safety practices. Once the Planning Stage is complete, the SMP moves into the Implementation Stage. In this stage, the SMP puts the written FSP into practice. The last stage is the Management Stage, in which the SMP must ensure that the written FSP is continually adhered to. We identify these three stages to help clarify the different activities and costs associated with each stage, but of course, in practice, there is an iterative aspect to any FSP plan as it is a dynamic document that needs to account for constant readjustments.

Fixed costs occur periodically and are independent of the quantity of product manufactured. Variable costs fluctuate according to how much product is manufactured. For example, the purchase of a scale is a fixed cost; it is purchased regardless of how much product is manufactured, while the number of jars purchased is a variable cost that depends on how many jars of the product are manufactured. In the following framework, we think in terms of a one-year time period. Recurring costs that can easily be estimated for a period of a year are considered to be fixed costs. Our focus in this research was to document fixed costs for existing SMPs, so we did not document most variable costs in this research. Examples of variable food safety costs include hair and beard nets, sanitizer, environmental swabs, and other items that are used in proportion to how many hours are spent on production, which itself is based on how much product is being manufactured. These costs were already incorporated into production costs by our participating SMPs, but we note that SMPs in the start-up phase of business development will need to consider variable food safety costs in addition to the fixed costs that we discuss below.

In addition to easily identifiable costs of producing plans, documents, and records that support SMPs’ FSP [21] there are “soft costs” associated with food safety. The existing literature on PCHF compliance often distinguishes between barriers such as “time”, “food safety culture”, “training”, and up-front capital expenditures and training fees typically associated with the cost barrier. A critical contribution of our research is to identify these “soft cost” barriers, measure them, and treat them as critical component of overall compliance costs. Soft costs are usually fixed costs associated with personnel, such as training, staff meetings, and *ad hoc* education regarding food safety culture. We track and identify the amount of staff time spent on planning, implementing, and managing food safety as critical compliance soft costs, even though they are difficult to pin down and measure. While this element is often omitted in cost estimation, it is well-documented that establishing a food safety culture involves costly changes in practice, commitment, establishing metrics, monitoring, and commitment to continuous improvement plans [22, 23].

Total cost of compliance for respondent *i* is presented in Eq 3, where *TCC*_*i*_ combines summed Planning Costs ∑*PC*_*i*1_ in year one, Implementation Costs ∑*IC*_*i*1_ in year one, and Management Costs $\sum MC_{it}^{t}$ in year *t*.

$$\begin{array}{c} {\text{TCC}}_{i}=\sum_{t=1}^{T}[\sum PC_{i1}+\sum IC_{i1}+\sum MC_{it}] \end{array}$$ (3)

This study does not explicitly analyze differences between costs for shared-use *versus* owned facilities due to small sample size. While we have 21 participants that *use* shared-use facilities, only two participants that operated shared-use facilities. Generally speaking, however, operators of shared-use facilities or established businesses already have GMP policies and sanitation standardized operating procedures (SSOP) that reduce the time to build food safety programming and help enable a food safety culture. Shared-use facility managers assume responsibility for planning, implementing, and managing facility-wide procedures, which can reduce costs for users of these facilities, but requires shared-use facility managers to assume some responsibility for enforcing facility guidelines with a wide and rotating population of businesses and employees that make a wide variety of products. Shared-use facilities generally pass on some portion of these costs to facility users through membership fees and rental rates which provide some financial support to alleviate the time and process burden of managing food safety compliance. Shared-use facility users, of course, must pay these fees. Users must also adhere to facility requirements, which can be more onerous than what the users’ product would necessitate due to the potential for cross-contamination.

Self-operated and new businesses must account for the time and effort to establish GMP and SSOP procedures, secure proper licensing (*i.e.* wholesale licensing) and permits necessary to establish a food production facility. These businesses must also invest in hiring and training employees and establishing a food safety culture.

## Results and discussion

We first compare changes in self-reported perceived knowledge of participants from before and after the full extension and technical assistance programming. Then we present the costs estimated from the Group Session participants. To ensure that our reported cost estimates do not inadvertently reveal sensitive financial information provided to us by our Group 1 and 2 participants, we report our summary statistics and results at the least granular level. Summary statistics are therefore reported for the largest group of participants: those that attended the Webinar.

### Summary statistics

**Fig 3:**
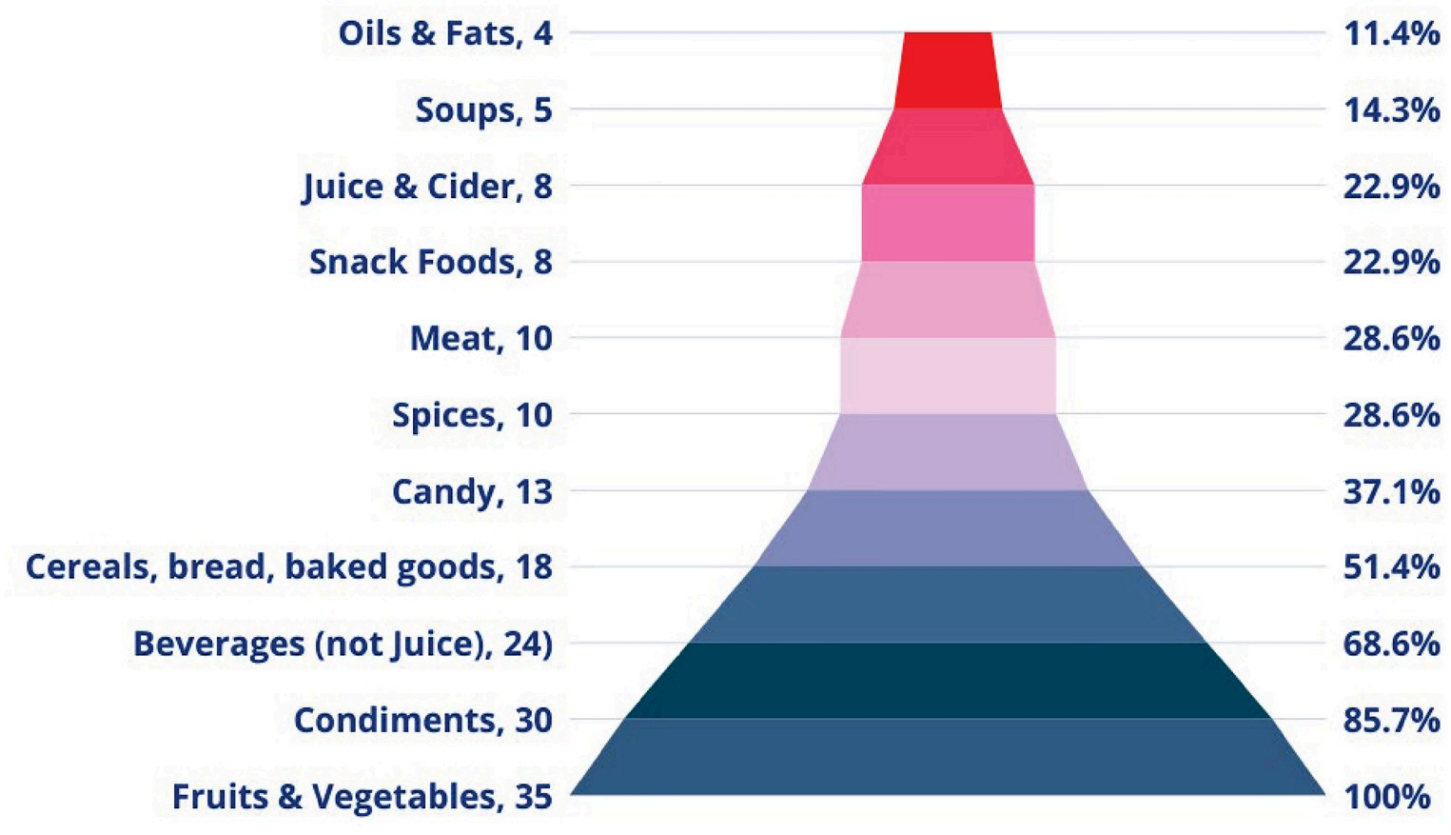
Products manufactured by participating SMPs.

Webinar participants manufactured a broad range of PCHF-covered products. Of the 81 webinar participants, most manufactured fruit and vegetable products (35), with condiments (30), non-juice beverages (24), and cereals (18) ranked second, third, and fourth. Fig 3 presents a funnel chart of products manufactured by the number webinar participants and percentage of each product type. We provide summary statistics at the webinar level only to protect the anonymity of our participants.

**Fig 4:**
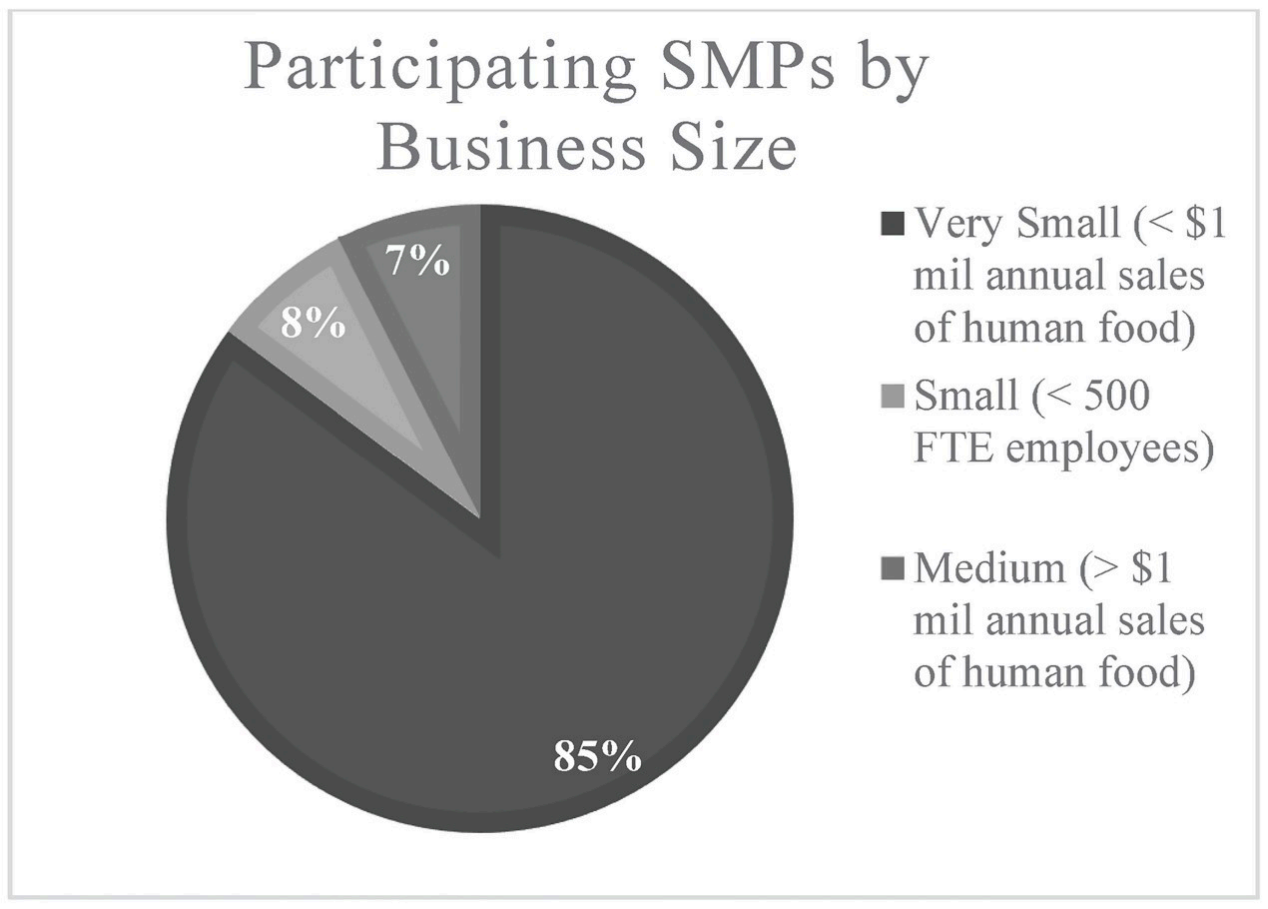
Participating SMPs by business size.

Most webinar participants (69) fell into the category of “very small” food processors, with less than $1 million in annual sales of human food per year (see Fig 4). The plurality of respondents had Gross Sales of $100,000 to $499,999 per year (see Table 2. Similarly, 75 participants had between 1 and 20 Full-Time Equivalent (FTE) employees (see Table 3. Participants manufactured products in a variety of facility types, including their own facility (33), shared-use kitchens (21), incubator kitchens (7), and home kitchens (5) (see Table 4).

**Table 2:** Gross sales of participating SMPs.

Gross Sales	Frequency	Percent
<$10k	16	19.75%
$10k-$24,999k	9	11.11%
$25k-$49,999k	8	9.88%
$50k-$99,999k	16	19.75%
$100k-$499,999k	19	23.46%
>=$500k	13	16.05%
Total	81	100%

**Table 3:** Number of full-time equivalent employees for participating SMPs.

Employees	Frequency	Percent
1 to 20	75	92.59%
21 to 49	5	6.17%
50 to 999	1	1.23%
Total	81	100%

**Table 4:** Facility type used by participating SMPs.

Facility Type	Frequency	Percent
Incubator Kitchen	7	8.86%
Shared-Use processing facility\commercial kitchen	21	26.58%
Business owns own facility	33	41.77%
Home kitchen	5	6.33%
Other	13	16.46%
Total	79	100%

We estimated FSP costs for ten SMPs that had participated in one of two Group Sessions (Group 1 n = 4; Group 2 n = 6). We calculated the hours spent by each SMP in the training series and included them as a “Planning Fixed Cost”. Averages in the following discussion are based on those SMPs that incurred the respective costs. Of the ten SMPs interviewed, four SMPs manufactured product in a shared-used facility, while six manufactured product in a stand-alone facility. Five of the SMPs manufactured fruit and vegetable products, three manufactured condiments/ sauces, one manufactured ready-to-eat snack products, and one manufactured dairy products.

### Changes in self-reported perceived knowledge pre-webinar to post-PCQI

Recall that we asked participants to identify their self-reported perceived knowledge of FSP planning and implementation assessment areas on a scale of 1–5, from “Not at all Knowledgeable” to “Extremely Knowledgeable”. In this paper, we primarily report unpaired unequal two-tailed T-tests of overall changes in self-reported perceived knowledge (scale of 1–5, from “Not at all Knowledgeable” to “Extremely Knowledgeable”) from the pre-webinar assessment to the post-PCQI assessment resulted in responses of (N = 81 and to N = 25, respectively). We note that there were no statistically significant baseline differences between pre-webinar levels of reported overall self-knowledge between the cohort of participants that completed the first three program units and the participants that did not continue with programming.

We reject the null 1 that programming did not result in changes in knowledge in most content areas. Reported knowledge increased within most content area topics (within non-food safety areas and food safety areas). For example, self-reported knowledge about costs of compliance increased by 1.3 points, on a 1-to-5-point Likert scale from before the educational programming (1.9) until after the PCQI course (3.2) (Pre-Webinar-Post PCQI, statistically significant at p>0.05). Average self-reported perceived knowledge for aggregate food safety content, however, increased by 1.4 points over the same period (from 2.7 to 4.1), and post-PCQI knowledge of costs was almost a full point lower than the average knowledge for food safety content areas (3.2 versus 4.1). This result indicates that there is room for improvement in PCHF training materials to include information about compliance costs, for the purpose of removing an important barrier to compliance that results from a lack of knowledge.

Like Henson and Heasman [8], Barone, DiCaprio, and Snyder [12] and Grover, Chopra, and Mosher [11], we find that, at the outset, the lowest levels of self-reported perceived knowledge among all assessment areas were for “Estimating costs of compliance and financial risk of non-compliance”. In Table 5 we assess self-reported perceived knowledge of costs among the 81 respondents who participated in a webinar, we find that, prior to programming, only about 6% of all pre-webinar participants reported that they were either “Extremely” or “Very” knowledgeable about compliance costs; about 25% indicated that they were “Moderately” knowledgeable, and 69% reported that they were either “Slightly” or “Not at all” knowledgeable about compliance costs (N = 81). Similarly, Harrison, Critzer and Harrison [24] found that lack of capital for training, a lack of understanding food safety laws specific to food processing were amount some of the leading barriers of Preventive Controls for Human Food compliance. Among the cohort of participants that continued programming through the PCQI course, these numbers were slightly different: about 8% of pre-webinar participants reported that they were either “Extremely” or “Very” knowledgeable about compliance costs; about 32% indicated that they were “Moderately” knowledgeable, and 60% reported that they were either “Slightly” or “Not at all” knowledgeable about compliance costs (N = 25).

**Table 5:** Changes in perceived knowledge from unpaired, unequal T-tests.

Knowledge Areas	Pre-Webinar		Post-PCQI		Change in Perceived Knowledge
	Mean	SD	Mean	SD	
**Non-Food Safety Areas**					
Audit	2.2	1.0	3.7	0.9	1.5*
PC Steps	2.6	1.0	4.3	0.6	1.7*
Cost	1.9	1.0	3.2	1.0	1.3*
*Average*	*2.2*		*3.7*		*1.5*
**Food Safety Areas**					
Food Risk	2.8	1.1	4.1	0.8	1.4*
FSMA	2.4	0.9	3.6	0.6	1.2*
Hazards	2.4	1.0	4.0	0.7	1.6*
Recall	2.7	1.2	4.0	0.7	1.4*
Verification	2.2	1.0	4.0	0.7	1.9*
Process Flow	2.5	1.1	4.2	0.7	1.7*
Product Description	2.6	1.0	4.2	0.6	1.6*
FSP Steps	2.8	1.0	4.2	0.6	1.5*
GMP	2.9	1.0	4.2	0.5	1.3*
GSP	3.7	0.9	4.3	0.6	0.6*
PC Rule	2.1	0.9	4.2	0.5	2.0*
Records	3.2	1.0	4.3	0.6	1.2*
*Average*	*2.7*		*4.1*		*1.4*
	N = 80		N = 25		

Note: Statistical significance denoted by *, from unpaired T-test with p>0.05. 5-point Likert scale from “Not knowledgeable at all” to “Extremely knowledgeable”.

**Fig 5:**
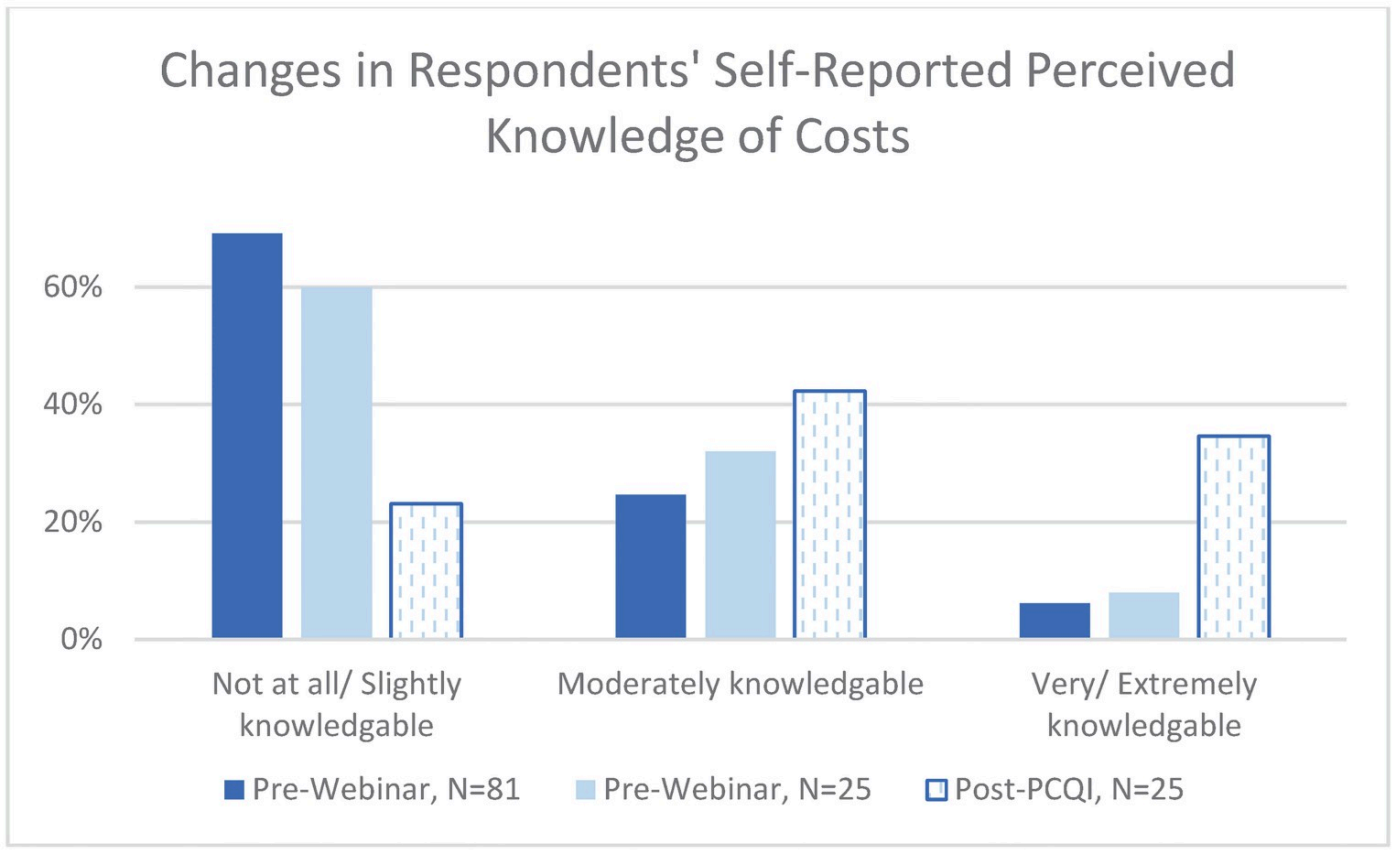
Changes in participants’ self-reported knowledge of costs, by cohort.

Interestingly, we did see increases in self-reported knowledge of costs over the course of the modified programming. Post-PCQI, about 35% of participants reported that they were either “Extremely” or “Very” knowledgeable about compliance costs; about 42% indicated that they were “Moderately” knowledgeable, and 23% reported that they were either “Slightly” or “Not at all” knowledgeable about compliance costs (N = 25, See Fig 5). While it is the case that the respondents who completed the PCQI course did have a slightly higher pre-webinar levels of knowledge regarding costs, the differences are not statistically significant, and are insufficient to attribute the changes in knowledge to a loss of less-knowledgeable participants over the course of programming S2 Table. We focus the remainder of our analysis on a comparison of the changes in knowledge between unpaired T-tests of overall changes in self-reported perceived knowledge from the pre-webinar assessment to the post-PCQI assessment (N = 81 to N = 25). Given the lack of explicit programming related to quantifying actual compliance costs, we might attribute this increase in knowledge to a result of exposure to clearly defined FSP tasks combined with SMPs’ experiential knowledge of corresponding costs. It is an important finding that merely acknowledging that FSP implementation incurs costs serves to increased perceived knowledge of food safety costs.

**Fig 6:**
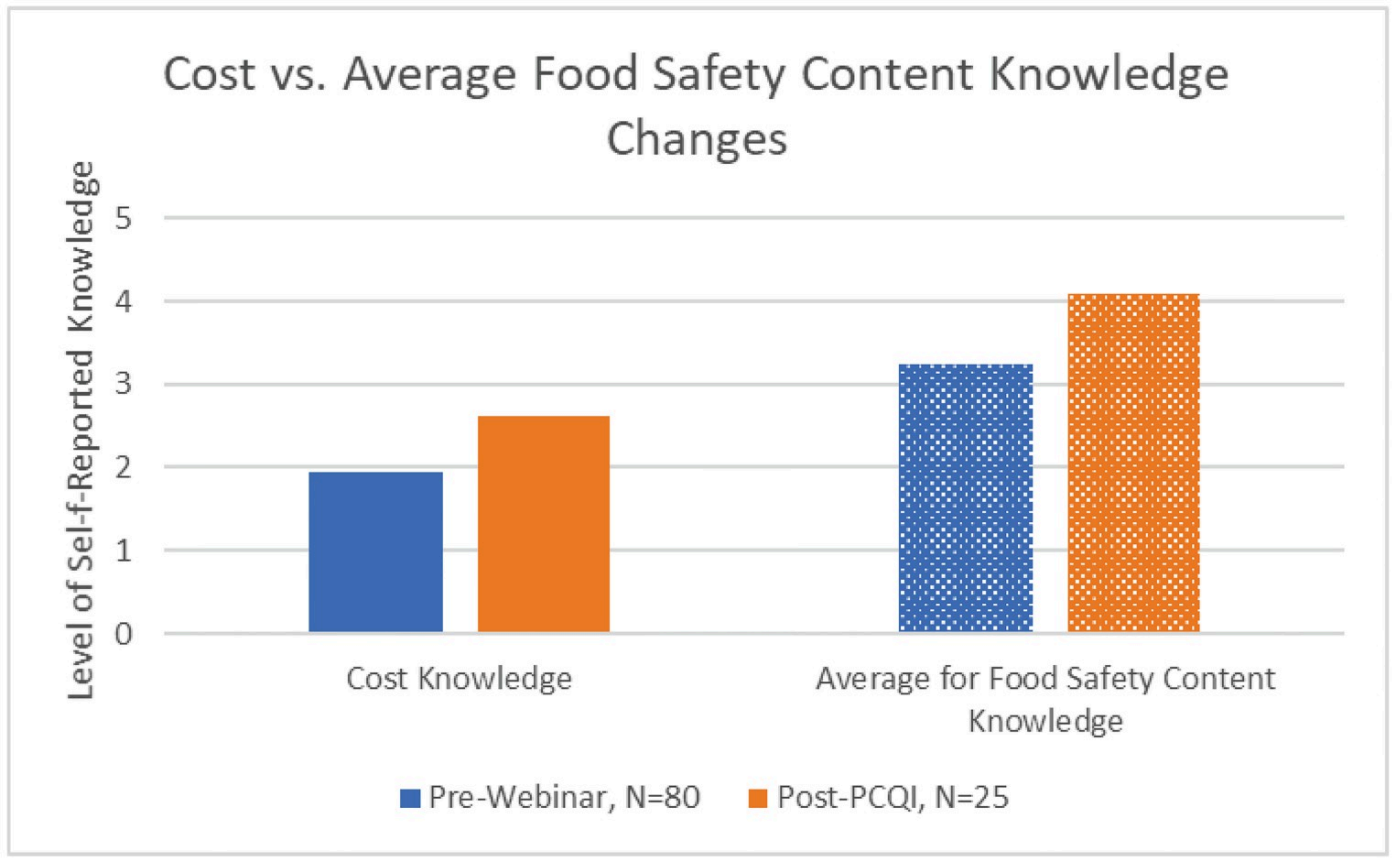
Knowledge changes for costs vs. average food safety content.

Despite the increase in knowledge about costs, this assessment category lagged behind all others in terms of the extent to which programming improved knowledge. Since the tiered FSP training and the modified curricula for the PCHF course only *acknowledged* that there are costs of compliance but did not seek to provide information regarding the costs themselves, it is perhaps not surprising that the learning area with the lowest mean learning, post-PCQI, remained costs of compliance. Fig 6 shows self-reported levels of knowledge prior to all extension programming and after the standard PCQI course. To provide context for understanding the relative change in pre-and post-changes in self-reported knowledge of costs, we provide average measures of pre- and post- changes in knowledge for all other food safety content categories.

### Costs of compliance tasks

Barriers to PCHF compliance that have been identified in the literature include categories such as “time”, “food safety culture”, and “training”. We refer to these “soft costs” and note that they are often related to personnel and are distinct from the up-front capital expenditures and training fees typically associated with the cost barrier. Upfront costs are relatively straightforward to estimate—there will be an invoice for a pest management company or a registration fee for a ServSafe^®^ training, for example. To identify soft-costs, we asked Group Session participants in our project to consider the compliance tasks they had implemented. We measured the costs of time that the participants spent in the different pieces of training and technical assistance that we provided, the time spent reviewing learning materials and applying those materials to their food product, and any other costs incurred as a result of pursuing PCHF compliance tasks and meeting standards. Notably, we only followed the costs as they accrued for a single product. For SMPs that manufacture multiple products, some of the costs will be incurred for each product manufactured. Despite that caveat, many of the costs we identify are “soft costs” that will provide benefits across individual products. Cost estimates are described below and reported in Table 6.

**Table 6:** Average costs table.

Planning							Average Costs per SMP
Average Soft Costs				Average Up-Front Costs			
Homework		Training					
Hours	Cost	Hours	Cost		Validation	Consultation	**$5,272**
33	$1,329	45	$1,749		$2,157	$2,250	
**Implementation**							
Average Soft Costs				Average Up-Front Costs			
Owner\Manager		Staff					
Hours	Cost	Hours	Cost	Equipment \Facilities	Audit \Inspection	Pest Control	**$8,731**
61	$2,358	43	$997	$11,500	$3,167	$1,867	
**Management**							
Average Soft Costs						Average Up-Front Costs	
Owner\Manager		Staff		Culture			
Hours	Cost	Hours	Cost	Hours	Costs	Training Certification Fees	**$7,929**
101	$3,560	98	$2,063	392	$6,007	$1,980	

Note: The term “Average” cost refers to averages across respondent SMPs who reported respective costs in this study. We did not measure quantity produced. This term diverges from the economic definition of “Average Costs”.

#### Planning stage

Personnel Costs make up the bulk of the fixed costs at the Planning Stage. Each participant in Group 1 spent about 49 hours, and each participant in Group 2 spent about 43 hours in training sessions with Food Safety Extension staff over the course of the program. We asked the SMPs to provide us with their hourly compensation and estimate that it cost, on average, $1,749 in labor costs per participant to attend the training.

In addition, on average, participants spent about 33 hours over the course of the training to complete “homework” assigned during training sessions to apply lessons learned to their own food business. On the low end, one SMP spent about 24 “homework” hours for a small, established business operating in a stand-alone facility with a low-hazard product. On the high end, two participants spent over 40 hours on “homework” reflecting larger staff, a shared-use facility, and/or products with higher hazards. We estimate that it cost, on average, about $1,329 on labor costs associated with completing the “homework”.

For Non-personnel fixed Planning Costs, six SMPs needed to hire external consultants to provide guidance on FSP planning, in addition to Extension services and training provided by the program. On average, the cost of external consultations was about $2,157 per SMP. Additionally, four SMPs needed to pay for external Product Validation. Validation refers to the scientific basis that justifies the process preventive control (if applicable). On average, the cost of validation was about $2,250 per SMP. In some cases, this cost included: a) service from a process authority to issue a scheduled process (*i.e.* for acidified shelf-stable foods); b) production trials to confirm the targeted internal temperature of the food at the coldest point could be reached; or, c) upfront investments for equipment or tools, such as a titrator to confirm sanitizer concentrations.

Potential additional costs that were not reflected in our participants’ needs include sanitation cost. Examples of sanitation costs include additional consultation to determine whether there is a danger of and how to contain cross-contamination (*e.g.* allergen residue or pathogens in ready-to-eat foods). Containment efforts would include training personnel (fixed cost), purchasing consumable supplies (variable costs), and fee-for-service at an outside microbial laboratory (fixed costs).

The Total Planning fixed costs averaged about $5,272 per SMP, with the highest at $12,165 and the lowest at $2,190 per SMP.

#### Implementation stage

Personnel Costs at the Implementation Stage vary quite a bit. On average, SMPs Owners and Managers spent about 61 hours implementing the plans they had developed in the Planning Stage. One SMP, however, only spent five hours implementing changes to an FSP that was very highly developed prior to the program, while a much newer business spent about 120 hours implementing an almost-new FSP from scratch. The average cost for the SMP owners/ managers’ labor at this stage was about $2,358. In addition, this is the stage at which some processors began to involve line staff in implementing the FSP. Of those SMPs that worked with staff to provide training and education at this stage, each spent about 43 labor hours at a cost of about $997 per SMP. Total Personnel Costs at the Implementation Stage ranged from $300 to $6,750 per SMP, with an average of $2,61 per SMP.

Non-personnel Implementation Costs include new investments in equipment or facilities (an average of $11,500 for the four SMPs); audit/inspection fees ($2,500 per audit or inspection); and pest control (an average of $1,867 for three SMPs).

Altogether, on average, SMPs spent about $8,731 on Implementation Costs, ranging from $300 to $27,800.

#### Managing stage

Managing fixed costs are, again, primarily a function of personnel costs. These costs can include ongoing food safety trainings and certifications for both owners/managers and hourly staff, regularly scheduled food safety mentions during staff meetings, new employee onboarding, and daily or even hourly engagement with the FSP to ensure best practices (such as monitoring activities, record verification, and reoccurring training). On average, owners and managers spent about 101 hours per year on managing, for an average of $3,560 per SMP per year. Additionally, staff spent on average 98 staff hours per year on managing, for an average staff cost of $2,063 per SMP per year.

In addition, SMPs included the cost of training and certifications in their estimates. The average SMP invests about $1,980 per year to send owners/managers and staff to food safety trainings. In addition, three of the SMPs invest, on average, $6,007 per year to carve out time to discuss the food safety culture of the business, including deeper dives into the “why” and “how” of the FSP.

Potential additional costs that were not reflected in our participants’ needs might be incurred for specific food products, depending on the type of product, process, and facility that is being used. For example, if processors identify a hazard during the hazard analysis, they must write, implement, and maintain ongoing records to maintain a monitoring activity. Additionally, a specifically trained person (PCQI) must review and verify the records and the other supporting documents (such as corrective actions, calibration logs, and training records) to confirm that the FSP is working as intended. Initial plan writing must be completed within 7 working days of the monitoring record and the FSP must be reviewed at least every 3 years or when a significant change occurs to the product or facility that would potentially introduce an unidentified hazard. This requires a team to be assembled and re-review the existing plan and make any necessary plan changes and/or record updates accordingly.

In total, on average, SMPs spend about $7,929 per year to Manage the FSP.

#### Total food safety costs

SMPs are right to consider the costs of food safety as among the important costs of doing business. In our study, we find that in the first year, SMPs should expect to spend about $21,932 to Plan, Implement, and Manage their FSPs, and they should expect to spend almost $8,000 in every subsequent year to Manage and enforce the FSP. These costs, of course, do vary according to the business itself, including what stage of life the business is at, the hazards involved in the product manufactured, how much product is manufactured, what kind of facility is used, etc. But the overall cost structure, particularly for a small or beginning food business, is important.

## Conclusion

In this paper, we investigate cost barriers that inhibit Small and Medium Processors to meet standards established in FSMA’s PCHF Rule. We seek to identify pathways to reduce the perceived cost barrier to compliance tasks associated with meeting these standards. We focus on SMPs that are “Qualified Exempt Facilities” as defined by FDA, and therefore are considered to be “in-compliance” with the federal regulation by completing a minimal list of compliance tasks and filing an attestation with the FDA, but that may still pursue additional compliance tasks (*e.g.*, pass a third-party audit). Through a series of educational programming units, we test the ability of current modified training tools to increase SMPs’ knowledge of compliance task costs. We find that, while merely acknowledging that SMPs will incur costs significantly increases participants self-reported knowledge of compliance task costs, knowledge of costs lags behind other knowledge areas. To address this deficit, we follow a cohort of SMPs through a rigorous, supported process of developing PCHF-compliant FSPs and evaluate the costs that they incur in that process. Notably, we identify and measure “soft-costs” associated with developing a PCHF-compliant FSP and develop a framework and estimate costs of compliance tasks for SMPs. We find that SMPs should initially expect to spend, on average, about $20,000 to Plan, Implement, and Manage their FSPs, and they should expect to spend almost $8,000 in every subsequent year to Manage and enforce the FSP.

Our findings affirm both theoretical propositions that lack of knowledge about costs poses a barrier to meeting regulatory standards [7, 8, 15, 25], and recent evidence that meeting food safety standards can be costly for small and medium sized businesses [18, 19, 26]. The weight of enforced food safety regulations falls on large processors, exempting small and medium processors from many compliance tasks that are intended to proactively reduce the incidence of foodborne illness. Since SMPs are exempt, these compliance tasks will only be completed voluntarily for this population. The perceived costs of performing these tasks is one of the most documented barriers to completion, but there is no formal training that educates SMPs about costs, in part because the costs were generally unknown and undocumented.

Our framework and cost results fill an important gap in the literature and provide the food safety community with concrete information to reduce perceived cost barriers and support SMPs to meet PCHF standards [27].

There are limitations to our results, including a small sample size, focus on the Northeast U.S., virtual interviews due to COVID-19 protocols, and reliance on self-reported data. We note that the participants drew from a current list of food processors in the Northeast U.S., and this study is not designed to be representative of the food industry as whole. As with most qualitative studies, the cost research in this study was designed to understand in-depth processes practiced by specific organizations. This sample included processors of various sizes and experience levels, therefore the background knowledge, learning outcomes and operational cost estimates may be influenced by this variability. Due to the COVID-19 pandemic, our planned in-person programming for the PCQI course and the Group Sessions was moved to virtual delivery. Since content delivery was entirely virtual, processors that seek in-person learning opportunities in the future may need to invest in travel costs. Other factors that could potentially sway overall cost structure could include business structure, location, and life-stage; product characteristics; idiosyncratic owner-operator preferences for risk, altruism, ambiguity, and other social outcomes, and owner-operator pre-knowledge. Despite these limitations, we develop a rigorous framework for analysis along with providing useful initial data that can inform future research.

The Preventive Controls for Human Foods Rules is poised to have a large impact on SMPs in the coming years, as the recognition of the role that SMPs plays in mid-scale supply chains increases [28]. As the federal government prioritizes regional-scale food production to mitigate global supply chain disruptions [29], the role of best food safety practices for small and mid-scale producers is likely to become of critical importance [28]. At the same time, changes that are being considered at the FDA may increase the urgency for SMPs to adhere to PCHF rules. In the fall of 2022, an Independent Expert Panel was convened to review FDA’s Human Foods Program and provide recommendations to strengthen the FDA’s regulatory role, as many of FSMA’s intended authority expansions had yet to be realized nearly a decade later [30]. Among the panel’s recommendations are those to strengthen existing PCHF requirements and align the consequences of violations of PCHF Rule with those of HACCP, as well as to obtain new authorities to invoke Civil Money Penalties for violations, including the failure to register [30].

FSMA requirements and standards impact all domestic U.S. food businesses directly and indirectly impact food businesses globally [1]. While the Preventive Controls for Human Food is a United States federal regulation, it leans heavily on HACCP-based concepts that are globally adopted to improve the safety of the food supply. Food businesses that import products into the United States must adhere to FSMA requirements, and growers, processors, and shippers must be prepared to satisfy the FDA’s increasing enforcement demands [11, 31].

In addition, food safety policies and regulations in developed food production countries, such as FSMA, affect food businesses across the globe, particularly as global supply chains become more intertwined [9]. While this study is a Northeastern-based study within the US, findings of this work are globally relevant as many small processors are challenged to comply with various food safety management systems. For example, Ru et al. [32] have found that small processors within a region in Italy had lower awareness and were hard-pressed to comply with dairy and meat production regulations due to similar limitations presented in this study. Similarly, smaller-scale dairy producers in Kenya were more likely to lack food safety programs for their facilities and fewer resources to help manage food safety controls [33]. Systems of food safety control around the globe have evolved to included “complex interaction between public and private modes of regulation” [15]. Downstream buyers are increasing requirements for compliance with complex food safety standards that include third-party audits or other voluntary certifications, regardless of the business’ status under government regulations. In the US, third-party audits could significantly reduce market access for QEF SMPs as these audit schemas traditionally require significantly more compliance tasks, such as written protocols, record keeping, training, and product analysis. Though third-party audits are not required for PCHF compliance, SMPs and suppliers are frequently asked to obtain a third-party audit to ensure overall safety of their facility. QEF SMPs often struggle when preparing, implementing, and managing compliance tasks for third-party audits [10, 12].

Going forward, it will be important for policy makers and food safety educators to reduce as many barriers as possible and provide SMPs with the information they need to be in compliance with FSMA’s PCHF Rule. Providing strategies to reduce cost barriers, one of the most significant barriers to compliance, is a critical step to achieve that goal. Findings from this study help inform policymakers and food safety educators about the barriers that impede SMP conformance to FSMA’s PCHF Rule. While our sample is small and confined to one region of the U.S., we believe that there are several findings that have direct relevance across all regions. Food safety educators would do well to consider aspects of compliance uptake that extend beyond awareness of regulations [13] and food safety content delivery [34]. Training materials that currently are limited exclusively to food safety content could be modified to include basic acknowledgements that costs are associated with food safety. To this end, food safety educators can use our methodology and baseline results to educate themselves about costs, and increase efforts to provide meaningful guidance to SMPs about costs. Future research can employ our framework and methodology to research costs for SMPs in other regions and with larger samples, potentially focusing on differences between sub-populations of SMPs (*e.g.*, shared-use *versus* self-owned facilities, new *versus* established businesses, small *versus* medium sized businesses).

To better support this cohort of processors, future strategies and support must account for the cost barriers to better enable compliance for SMP.

## Supporting information

S1 Fig Hensen and heaseman model of compliance. The model identifies nine stages of the compliance process [8].
(TIF)

S1 File Food safety modernization act Preventive Controls for Human Foods. A description of PCHF requirements and standards.
(PDF)

S1 Appendix Qualitative research. Qualitative study design.
(PDF)

S2 Appendix Expanded literature review of compliance cost burdens. Expanded literature review of compliance cost burdens.
(PDF)

S1 Table Knowledge evaluation areas. (PDF)

S2 Table T-tests of differences between pre-webinar baseline self-reported knowledge by cohort. (PDF)
