# Supplementary figures and images for: Assessing the cost barrier for small and medium food processing businesses to meet Preventive Controls for Human Foods standards
Source: PLoS One. 2024 Sep 13;19(9):e0306618. doi: 10.1371/journal.pone.0306618 (PMC11398691; doi:10.1371/journal.pone.0306618)

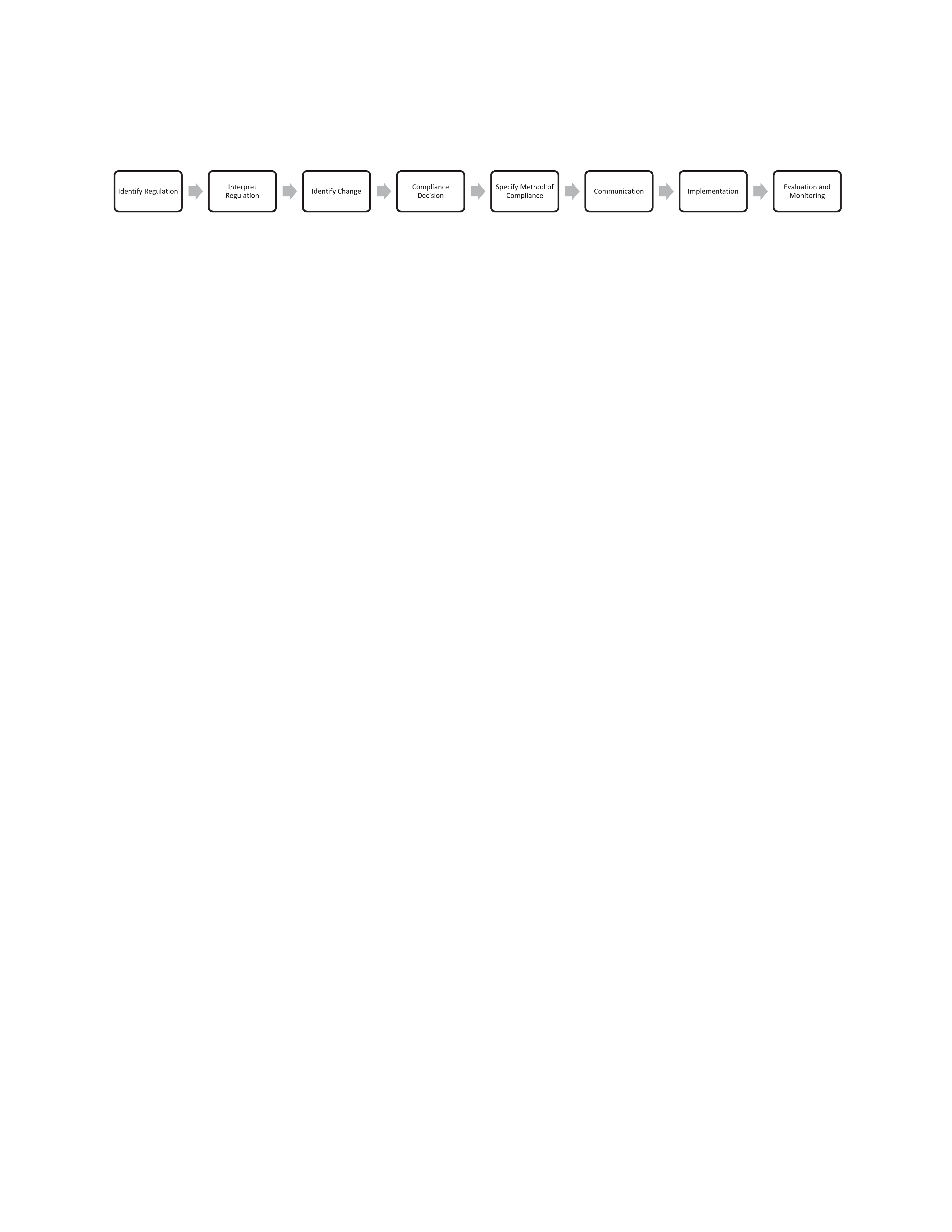

Supplement: S1 Fig — The model identifies nine stages of the compliance process [8]. (TIF) [file pone.0306618.s001.tif]
