# Supplementary material for: Assessing the cost barrier for small and medium food processing businesses to meet Preventive Controls for Human Foods standards
Source: PLoS One. 2024 Sep 13;19(9):e0306618. doi: 10.1371/journal.pone.0306618 (PMC11398691; doi:10.1371/journal.pone.0306618)
Supplement: S1 File — A description of PCHF requirements and standards. (PDF) [file pone.0306618.s002.pdf]

S1 File. Food Safety Modernization Act Preventive Controls for Human Foods.

A description of PCHF requirements and standards.

The Food Safety Modernization Act Preventive Controls for Human Foods rule includes updated cGMPs and new requirements for identifying and managing the process, sanitation, food allergen, and supply chain preventive controls for certain processed foods not already covered by other United States federal food safety regulations, such as juice, seafood, and USDA-meat and poultry that require Hazard Analysis of Critical Control Points (HACCP) food safety plans.

We note that the “Qualified Exempt Facilities” regulatory category defines businesses by their relationship to FSMA. Very few small businesses are likely to define themselves in this way, so we instead consider our population of interest to be “SMPs”, as small and medium sized food processors are more likely to self-identify in this way.
