# Supplementary material for: Assessing the cost barrier for small and medium food processing businesses to meet Preventive Controls for Human Foods standards
Source: PLoS One. 2024 Sep 13;19(9):e0306618. doi: 10.1371/journal.pone.0306618 (PMC11398691; doi:10.1371/journal.pone.0306618)
Supplement: S1 Appendix — Qualitative study design. (PDF) [file pone.0306618.s003.pdf]

S1 Appendix. Qualitative Research Study Design.

In this Appendix we outline the qualitative research design and how it addresses Domains identified by Tong [1].

Domain 1: Research team and reflexivity.

1. Personal characteristics
   (a) Dr. Fitzsimmons primarily conducted interviews, with assistance from Ms. Kinchla and Ms. Allingham.
   (b) Dr. Fitzsimmons holds a PhD in economics and focuses on business decision-making. Ms. Kinchla has an M.S. in Food Science and works with food businesses to provide food safety Extension and Training.
   (c) Dr. Fitzsimmons is a Research Assistant Professor. Ms. Kinchla is an Extension Assistant Professor. Ms. Allingham is a Graduate Assistant.
   (d) All interviewers are female.
   (e) Dr. Fitzsimmons has conducted several interviews and focus groups with small businesses regarding financial decision-making and firm financial structure.
2. Relationships with participants
   (a) Relationships with participants were established over a roughly eight-month period during which intensive training was provided to participants in both group and individual sessions.
   (b) Participants were informed of researcher goals.
   (c) Participants were informed of background of each individual researcher and what led each to their interest in this research.
   (d) Participants were informed of research motivations, research goals, use of research results, potential impact of research results.

Domain 2: Study design.

1. Theoretical framework
   (a) Our theoretical framework and methodology most closely follows content analysis. We began with a discussion of economic principles of firm costs, and then provided a spreadsheet format of costs that we had identified as researchers. We asked participants to both critique and revise the framework as a group and provide numeric estimates of items within the framework/revised framework as individual firms.
2. Participant selection
   (a) We used purposive selection to identify participants. In addition to selecting businesses that fell within our population of interest, criteria used included products manufactured, business life-stage, type of facility, stage of food safety preparedness, and food safety plan goals. We sought to have a broad range of participants with respect to these characteristics. Due to limitations presented by COVID 19, we also considered location.
   (b) Participants were approached over email.

(c) Four SMPs in Group 1 and six SMPs in Group 2.

(d) On SMP dropped out of Group 1 due to time commitment.

3. Setting

   (a) Data were collected online over Zoom meetings due to COVID 19.

   (b) Only participants and researchers were present.

   (c) All reportable demographics are available in the main body of the manuscript.

4. Data Collection

   (a) We began with a discussion of economic principles of firm costs, and then provided a spreadsheet format of costs that we had identified as researchers.

   (b) Repeat interviews were not carried out.

   (c) The research used video recordings to collect data as back-up.

   (d) Field notes were taken, primarily in the form of modifications to the spreadsheet and entry of numeric data.

   (e) Each interview session lasted between 1.5 and 2 hours.

   (f) Data saturation was not discussed.

   (g) Raw transcripts were not returned, but draft analyses and draft outputs were returned to participants prior to public release for comment/correction.

Domain 3: Analysis/findings

1. Data analysis

   (a) Numeric data were not coded. Two researchers reviewed numeric data and framework.

   (b) Themes were not identified, although an alternative framework that used economic theory to identify cost patterns was developed.

   (c) Microsoft Excel was used to manage and analyze data.

   (d) Participants provided feedback on findings.

2. Reporting

   (a) Quotations were not presented.

   (b) There is consistency between data and findings.

   (c) Major cost patterns within the framework are identified in findings.

   (d) There are descriptions of variations from primary cost patterns within the framework.

References

1. Tong A, Sainsbury P, Craig J. Consolidated criteria for reporting qualitative research (COREQ): a 32-item checklist for interviews and focus groups. International Journal for Quality in Health Care. 2007;19(6):349–357. doi:10.1093/intqhc/mzm042.
