# Supplementary material for: Assessing the cost barrier for small and medium food processing businesses to meet Preventive Controls for Human Foods standards
Source: PLoS One. 2024 Sep 13;19(9):e0306618. doi: 10.1371/journal.pone.0306618 (PMC11398691; doi:10.1371/journal.pone.0306618)
Supplement: S2 Appendix — Expanded literature review of compliance cost burdens. (PDF) [file pone.0306618.s004.pdf]

S2 Appendix. Expanded literature review of compliance cost burdens.

Henson and Heasman point out, that “since benefits are generally more difficult to quantify than costs, there will tend to be an in-built bias towards relatively higher perceived costs” [1]. Businesses are therefore more likely to expect that the costs of compliance are higher than the benefits, thus making decisions on the margin not to implement food safety measures. Mensah and Julien [2] identify the high cost of development and implementation as one of the top five challenges (26%) hindering compliance of enterprises to Food Safety Management Systems in the U.K. Yapp and Fairman [3] found that small and medium food enterprises in the UK perceived money (20%) and time (54%) to be barriers to compliance with food safety regulations in addition to lack of knowledge, motivation, and trust in food safety authorities. The negative aggregate impacts of the cost barrier of food safety compliance can reduce competitiveness of the sector, and have been considered to be serious enough to constitute a non-tariff barrier to trade [4].

More recently, Grover, Chopra and Mosher [5] analyzed Midwestern processors' perceived significance of challenges to FSMA PC implementation and found that cost of implementation was the second most significant challenge, due to cost of infrastructure investments, third party consultants, and employee training. Barone, DiCaprio and Snyder [6] similarly found that “cost” (19%) and “time” (25%) were the most frequently identified barriers for processors in Ohio to implementing ongoing employee food safety training for PCHF. The FDA itself acknowledges that small food processors face cost barriers to implementing PCHF [7]. A survey of Food Safety Educators in the Northeast found that barriers related to learning Food Safety Content consistently ranked lower than costs-related barriers. For example, on a Likert scale from “Extremely Challenging” to “Not Challenging at all”, the most challenging barrier is “costs for hiring additional workers to manage a food safety program”, which 44% scored as “Extremely Challenging” (44%) [8]. Barriers such as “lack of time for training” (25% Extremely Challenging) and “costs of training and enforcing compliance with current employees” (19% “Extremely Challenging”) outranked other food safety content-related barriers. This suggests that there is a gap between what SMPs' “perceive”, or fear, the costs of compliance may be, and evidence-based knowledge of what the costs actually are, indicating that the cost barrier may be about more than the actual financial barrier of a compliant FSP.

References

1. Henson S, Heasman M. Food safety regulation and the firm: understanding the compliance process1Financial support from the Ministry of Agriculture, Fisheries and Food (MAFF) is acknowledged. However, the views expressed in this paper reflect those of the author and not necessarily those of MAFF.1. Food Policy. 1998;23(1):9–23. doi:10.1016/S0306-9192(98)00015-3.

2. Mensah LD, Julien D. Implementation of food safety management systems in the UK. Food Control. 2011;22(8):1216–1225. doi:10.1016/j.foodcont.2011.01.021.

3. Yapp C, Fairman R. Factors affecting food safety compliance within small and medium-sized enterprises: implications for regulatory and enforcement strategies. Food Control. 2006;17(1):42–51. doi:10.1016/j.foodcont.2004.08.007.

4. Henson S, Caswell J. Food safety regulation: an overview of contemporary issues. Food Policy. 1999;24(6):589–603. doi:10.1016/S0306-9192(99)00072-X.

5. Grover AK, Chopra S, Mosher GA. Food safety modernization act: A quality management approach to identify and prioritize factors affecting adoption of preventive controls among small food facilities. Food Control. 2016;66:241–249. doi:10.1016/J.FOODCONT.2016.02.001.

6. Barone NA, DiCaprio E, Snyder AB. A preliminary assessment of food safety training needs and preferences among Ohio food processors of various sizes. Food Control. 2020;114:107220. doi:10.1016/J.FOODCONT.2020.107220.

7. Bass G. Keynote presentation. [2021] Available from: https://www.ifsh.iit.edu/sites/ifsh/files/departments/fspca/pdfs/2021-FSPCA-Virtual-AC-Information-Packet-and-Agenda_2021-10-19.pdf.

8. Fitzgerald A, Gilbert-Eckman A, Demmings E, Fitzsimmons JA, LaBorde L, Kinchla A, et al. Understanding the Food Safety Needs of Small and Very Small Processors in the Northeast United States: Food Safety Communicator and Regulator Perspectives Food Protection Trends. Food Protection Trends. 2024;Forthcoming.
