# Supplementary material for: Assessing the cost barrier for small and medium food processing businesses to meet Preventive Controls for Human Foods standards
Source: PLoS One. 2024 Sep 13;19(9):e0306618. doi: 10.1371/journal.pone.0306618 (PMC11398691; doi:10.1371/journal.pone.0306618)
Supplement: S1 Table — (PDF) [file pone.0306618.s005.pdf]

S1 Table. Knowledge Evaluation Areas.

Knowledge Evaluation Areas
Non-Food Safety Areas
Estimating costs of compliance and financial risk of non-compliance
The steps that I need to take within my facility to comply with Preventive Controls
3rd Party Auditing
Food Safety Areas
Drawing a process flow diagram
Conducting a Hazard Analysis
Identify verification activities
Record Keeping
The steps involved in a Food Safety Plan
Developing a recall plan
The Preventive Controls Rule
The FDA's Food Modernization Act
Good Manufacturing Practices
Good Sanitation Practices
Writing a product and process description

Respondents instructed to identify how knowledgeable they are about the topic areas on a Likert scale from 1-5.
