# Supplementary material for: Assessing the cost barrier for small and medium food processing businesses to meet Preventive Controls for Human Foods standards
Source: PLoS One. 2024 Sep 13;19(9):e0306618. doi: 10.1371/journal.pone.0306618 (PMC11398691; doi:10.1371/journal.pone.0306618)
Supplement: S2 Table — (PDF) [file pone.0306618.s006.pdf]

S2 Table. T-Tests of Differences between Pre-Webinar Baseline Self-Reported Knowledge by Cohort.

Perceived Knowledge Areas	Webinar Only		Webinar to PCQI			
	Mean	St. Dev.	Mean	St. Dev.	*t*	*p*
**Non-Food Safety Areas**						
Audit	2.2	1.0	2.2	1.1	-0.1	0.9
PC Steps	2.5	1.0	2.8	0.9	-1.1	0.3
Cost	2.0	1.0	1.9	0.9	0.5	0.6
**Food Safety Areas**						
Food Risk	2.7	1.2	2.8	0.8	-0.3	0.7
FSMA	2.3	0.9	2.6	0.9	-1.0	0.3
Hazards	2.4	1.0	2.3	1.0	0.3	0.8
Recall	2.6	1.2	2.9	1.4	-0.8	0.4
Verification	2.2	1.0	2.1	0.9	0.0	1.0
Process Flow	2.5	1.2	2.4	1.1	0.5	0.6
Product Description	2.6	1.1	2.6	1.0	0.2	0.9
FSP Steps	2.6	1.0	3.1	1.0	-1.8	0.1
GMP	2.9	1.1	2.9	1.0	0.2	0.8
GSP	3.6	0.9	3.8	0.9	-0.8	0.4
PC Rule	2.1	1.0	2.0	0.7	0.4	0.7
Records	3.2	1.1	3.1	0.7	0.1	0.9
	N=59		*N=21*			

Note: Statistical significance denoted by *, resulting from unequal t-test with p>0.05.
